# Transactions of the American Medical Association, Vol. IX. 1856

**Published:** 1857-02

**Authors:** 


*Transactions of the American Medical Association, Vol. IX.* 1856. Philadelphia: Printed for the Association by T. R. & P. G. Collins.

(Continued from Page 86.)

The next paper in the Volume of Transactions is the 'Report on the Causes which Impede the Progress of American Medical Literature,' by S. D. Gross, M.D. and Prof.

It occupies twenty-six pages, and is worthy of a careful perusal. In regard to the specific causes which are alleged as impediments to the progress of our medical literature, Dr. Gross makes the following summary:—

'1. The identity of the language of this country with that of Great Britain. 2. A disposition in the Profession to patronize English works in preference to American. 3. A want of independence in our periodical press. 4. A lack of industry in observing and recording facts in private and hospital practice.'

These topics are considered at length, and ith the well-known ability of the author. We are satisfied, however, that he has placed altogether too much stress on the *second* and *third* topics just enumerated.

That there is any disposition, either 'felt or evinced by the Profession, to patronize foreign works, especially English, in preference to our own,' we do not believe. On the contrary, we have very rarely met with a member of the Profession, whether as student or practitioner, who, if he was about to purchase a work on any department, did not give preference to American authors whenever any such existed possessing even a moderate claim to consideration. Neither do we believe that the teachers in our medical colleges possess any such predilection for *foreign* works as represented by Dr. Gross. Certain it is, that twenty years since, when we were in the lecture-rooms of our Alma Mater, more than half of the text-books in use were American. We had for text-books, Wistar and Horner on Anatomy; Beck on Chemistry; Eberle on Practice; Dewees on Obstetrics and Diseases of Females; and Eberle's Therapeutics; leaving only Physiology and Surgery to be supplied by foreign authors, and even on the first of these, Dunglison's work was holding a fair competition with those of Richeraud and Majendie. That foreign works on Physiology, Pathology, Animal Chemistry, and such other minor departments as require for their elucidation much closet study or direct experimental research, hold a predominant position, is undoubtedly true, and for reasons which will be obvious to every reflecting mind. But in the departments of Practical Medicine, Therapeutics, and Materia Medica, Obstetrics, &c. our own writers have been largely in the ascendant, both in the colleges and the libraries of the practitioners—as the works of Dewees, Chapman, Eberle, Wistar, Horner, Beck, Wood & Bache, Wood, Bell, Dixon, &c. abundantly prove. That some teachers as well as practitioners may have been careless in their selection of medical authors, or may have been actuated by a pedantic feeling of contempt for home productions, and a desire to appear learned in foreign literature, may be true, but we think Dr. Gross has exaggerated the evil.

Concerning the critical or review department of our medical periodicals, Dr. Gross gives the following description:—

'But it cannot be denied that, in the department of criticism, they are generally deficient in boldness, force, and judgment, falling far below the common standard in the same branch of literature in Great Britain. The reviews are, with few exceptions, written without taste and without point, as if their authors were afraid lest they should be accused of unkindness, harshness or ill-nature. They are characterized more by politeness than by a manly and independent tone, which is not afraid to utter its real sentiments and to affix the seal of its unbiassed judgment, They are marked by none of the masculine vigor which is so well calculated to impart zest to the reader, and cause him to regret that he is so near the end of his task; which infuses life and spirit into a journal, and makes it a welcome guest at the table of the physician; which fashions and directs the dart, but blunts its point before it is permitted to strike its victim; which metes out equal justice to all men, who come within its vitalizing, soul-stirring influence; which blends mercy with severity; which, when occasion requires, wounds but does not kill. It is a criticism which is neither alkaline nor acid, nor yet wholly neutral, but so nearly neutral as to render it impossible to determine its real character. It is a jesting, good-natured criticism, which, for fear of doing mischief, or of being thought unkind, is bound in swaddling clothes, lest by its sudden and inadvertent jokes it should kick over the milk and water in the inkstand of the happy, self-complaisant reviewer. In fine, it is an inert, a tame, a spiritless criticism; a criticism without body, without strength, without soul, deaf and dumb, and blind and halt.'

This exceedingly defective method of criticism the writer attributes to a 'lack of independence' on the part of editors, and a venal fear of offending the Book Publishers. In this we think he is altogether mistaken. We do not believe there are six editors of medical journals in the whole Union who ever pause to think whether the notices they write will ever be seen or read by the book publishers or not. Most of them are active practitioners, and some both practitioners and teachers. Their time is so closely occupied that it is impossible for them to read each volume as they receive it with that care which is necessary for a sound critical review: the consequence is, that, in nine cases out of ten, they read the title-page and table of contents, glance at here and there a page through the work, and make up a brief milk-and-water notice, sometimes eked out with a quotation or two, and by that time the 'Printer's Devil' is after them for *more copy*, and it is sent to the press. The true reason why the review department in American medical periodicals is so defective, is to be found not in the want of *independence* on the part of the editors, but in the fact that the work of medical journalism in this country is mostly gratuitous, and consequently done as collateral or supplementary to all the duties of an active professional life.

The fourth impediment to the progress of American medical literature, mentioned by Dr. Gross, consists in 'the little use that is made of the advantages afforded by private and hospital practice.' His comments on this topic are eminently just and appropriate. We ask our readers to consider carefully the following paragraph, which we quote from pages 359–'60–'61:—

'Every physician, however slender his talents or limited his opportunities, has it in his power to make himself useful to his profession. It is only necessary that he should carefully observe and faithfully record the facts that pass daily, for fifteen, twenty, or twenty-five years, under his eye to enable him to become a most valuable contributor to medical science and medical literature. If this habit were universal, the profession, and mankind at large, would not now have to lament the many imperfections and the many incongruities of the healing art. Many diseases which now baffle the skill of the physician and attest his impotence, would be rendered amenable to his remedies, and cease to be regarded as opprobious. And what is true of individual observation and experience, is still more true of combined observation and experience, those compound pulleys and levers of the human mind. Our country is rich in medical charities, hospitals, almshouses, infirmaries, and asylums of all kinds.'

'Some of the hospitals of our country have been in successful operation for upwards of a century, and yet, during all this time, they have literally been as sealed books to the bulk of the profession. The only light that has ever emanated from any of them has been an occasional ray, apparently grudgingly bestowed, in the form of a contribution to some medical journal, more transient, perhaps, than the journal itself. We might, if it might not seem personal, point to some of these establishments where materials for the study of pathological anatomy abound that even a Rokitansky might envy; to some, where vast opportunities are constantly afforded for the study of all kinds of injuries, as wounds, fractures, and dislocations; to some, where syphilis might be investigated, in all its forms and phases, with the same facility and amplitude as at the Hopital du Midi, in Paris; to some, where there are annually upwards of seven hundred cases of parturition, and any amount and variety of diseases of women and children; to some, where pulmonary, gastric, and intestinal affections are of constant occurrence; and, finally, to some, where eye and ear diseases are studied and treated as specialities.

'Of the one hundred and twenty thousand patients who, we have supposed, are annually admitted into the various hospitals, asylums, and other charitable institutions of the country, at least ten thousand die. The bodies of many of these are doubtless examined, but where are the records of the results? I am not aware that one solitary great and important paper on pathological anatomy has ever appeared in our medical journals from the pen of a hospital physician, surgeon, or accoucheur.

'The preceding facts require no comment: they speak for themselves. The patriotic physician, patriotic in a double sense, patriotic to his profession and to his country, may we exclaim as he contemplates these things, "Watchman, what of the night?" When will official station and opportunities be turned to account? When will the light of medical science be made to emanate from these institutions, and to shed its quickening and exhilarating influence abroad upon the medical profession and the world? Had the opportunities above alluded to been properly employed, how rich might our profession now be in great works on pathological anatomy, medicine, surgery, and obstetrics! What light might we not now send by every steamer to Europe in liquidation of our literary debt! Our foreign bonds would soon be cancelled, and American repudiation would cease to be a by-word among our transatlantic brethren.'

The following resolutions are appended to the report:—

'*Resolved*, That this Association earnestly and respectfully recommend, first, the universal adoption, whenever practicable, by our schools, of American works as text-books for their pupils; secondly, the discontinuance of the practice of editing foreign writings; thirdly, a more independent course of the medical periodical press towards foreign productions, and a more liberal one towards American; and, fourthly, a better and more efficient employment of the facts which are continually furnished by our public institutions for the elucidation of the nature of diseases and accidents, and, indirectly, for the formation of an original, a vigorous, and an independent national medical literature.

'*Resolved*, That we venerate the writings of the great medical men, past and present, of our country, and that we consider them as an important element of our professional and national glory.

'*Resolved*, That we shall always hail with pleasure any useful or valuable works emanating from the English press, and that we shall always extend to them a cordial welcome as books of reference, to acquaint us with the progress of legitimate medicine abroad, and to enlighten us in regard to any new facts of which they may be the repositories.'

A notice of the remaining papers in the volume must be deferred until our next issue. In the mean time let our readers forward *three* dollars either to Dr. D. Miller, of this city, or directly to the Treasurer Dr. Caspar Wistar, of Philadelphia, and procure a copy for themselves.

(To be Concluded in our next No.)

